# Mulberry Fruit Extract Protects Pancreatic β-Cells against Hydrogen Peroxide-Induced Apoptosis via Antioxidative Activity

**DOI:** 10.3390/molecules19078904

**Published:** 2014-06-26

**Authors:** Jong Seok Lee, Young Rae Kim, Jun Myoung Park, Suk-Jin Ha, Young Eon Kim, Nam In Baek, Eock Kee Hong

**Affiliations:** 1 Department of Bioengineering and Technology, Kangwon National University, Chuncheon 200701, Korea; E-Mails: jongseoklee78@gmail.coM (J.S.L); youngraekim0709@gmail.com (Y.R.K.); three0313@nate.com (J.M.P.); sjha@kangwon.ac.kr (S.-J.H.); 2 Korea Food Research Institute, Seongnam 463746, Korea; E-Mail: radog@kfri.re.kr; 3 Department of Oriental Medicinal Materials and Processing, Kyung Hee University, Youngin 446701, Korea; E-Mail: nibaek@khu.ac.kr

**Keywords:** mulberry, oxidative stress, apoptosis, diabetes, pancreatic β-cells

## Abstract

Among the many environmental stresses, excessive production of reactive oxygen species (ROS) and the ensuring oxidative stress are known to cause significant cellular damage. This has clinical implications in the onset of type 1 diabetes, which is triggered by the destruction of pancreatic β-cells and is associated with oxidative stress. In this study, we investigated the protective and antioxidative effects of mulberry extract (ME) in insulin-producing pancreatic β-cells. We found that ME protects pancreatic β-cells against hydrogen peroxide (H_2_O_2_)-induced oxidative stress and the associated apoptotic cell death. ME treatment significantly reduced the levels of H_2_O_2_-induced 2-diphenyl-1-picrylhydrazyl (DPPH) radicals, and lipid peroxidation and intracellular ROS accumulation. In addition, ME inhibited DNA condensation and/or fragmentation induced by H_2_O_2_. These results suggest that ME protects pancreatic β-cells against hydrogen peroxide-induced oxidative stress.

## 1. Introduction

Diabetes is one of the major metabolic diseases among adults in the developed world. Type 1 diabetes is classified as insulin-dependent diabetes mellitus, whereas Type 2 is defined as non-insulin-dependent diabetes mellitus [1]. Type 1 diabetes is caused by the loss of insulin secretion due to the destruction of pancreatic β-cells [2]. This leads to increased blood sugar levels, as well as the suppression of energy transfer to other organs, resulting in various secondary complications [3,4]. Therefore, the protection of pancreatic β-cells is paramount for preventing the onset of Type 1 diabetes. Under homeostatic conditions, potentially toxic reactive oxygen species (ROS) are primarily generated by mitochondrial respiratory metabolism, and are then efficiently neutralized by cellular antioxidant defense mechanisms [5]. However, excessive generation of ROS, such as superoxide anions (O_2_^−^) and hydrogen peroxide (H_2_O_2_), during times of environmental stress results in significant oxidative damage to cell structures such as DNA, mitochondria, and cell membranes. The oxidative stress caused by accumulation of ROS induces β-cell apoptosis, lipid peroxidation, and DNA condensation and/or fragmentation [6]. 

Recent studies have demonstrated that antioxidants can protect cells by reducing hydrogen peroxide-induced oxidative stress [7], *N*-acetylcysteine (NAC) is an antioxidant known to protect pancreatic β-cells by scavenging ROS *in vitro* and *in vivo*, respectively [8,9]. This indicates the importance of isolating substances with antioxidative activity and characterization of their antioxidant mechanisms. Plants have the ability to synthesize a wide variety of chemical compounds with important biological effects, including antioxidative activity [10]. Many of these plant-derived active compounds have beneficial long-term health effects when consumed by humans and are commonly employed in developing countries for the treatment of various human diseases [11,12]. Mulberry is widely distributed in Asia, Europe, America, and Africa. Korean mulberry (*Morus alba* L.), a member of the Moraceae family, has been primarily used for feeding silkworms (*Bombyx mori* L.), which eat its leaves and spin cocoons [13]. Mulberry is a well-known medicinal plant and has been used historically for suppressing edema, eliminating hangover and disease associated with thirst, among others [14]. Recent studies have suggested that pigments isolated from mulberry may contribute to its reported anti-aging and anti-hyperlipidemia properties [15,16]. There are many studies regarding phytochemicals and pharmacological effects of the leaves or roots of *Morus alba* [17,18,19,20]. Some studies have reported that mulberry fruit pigment has protective effects against oxidative stress in streptozotocin-induced diabetic rats or high fat diet-induced obese mice via antioxidant antioxidative defence systems [21,22,23]. In addition, a polysaccharide purified from mulberry fruits stimulates murine macrophages to release chemokine and pro-inflammatory cytokines [24]. However, its impact on oxidative stress-induced pancreatic β-cells death has not been investigated. In this study, we have investigated the protective effect of mulberry extract (ME) on hydrogen peroxide-induced oxidative damages in pancreatic MIN6N β-cells.

## 2. Results and Discussion

### 2.1. DPPH Radical Scavenging Activity of ME

**Figure 1:**
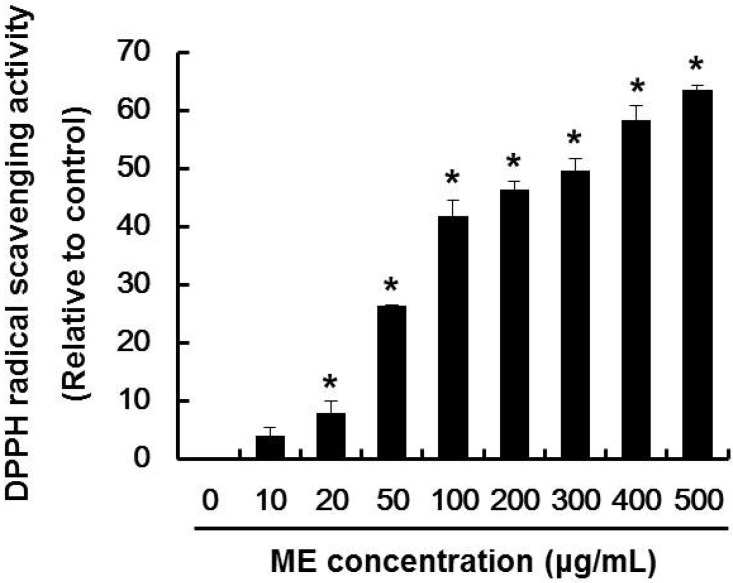
DPPH radical scavenging activity of ME. The amount of DPPH radicals was determined spectrophotometrically at 520 nm. Data represent the mean ± SE of three independent experiments. Significance of the differences were compared with the non-treated group at * *p* < 0.05 by Student’s *t*-test.

Deficiency of insulin caused by the destruction of pancreatic β-cells induces hyperglycemia, which leads to diabetes and serious pathological effects in humans [25]. Reactive oxygen species (ROS) are heavily implicated in the mechanism of pancreatic β-cells destruction. Thus, inhibition of ROS generation is an important therapeutic goal. In this regard, antioxidants play a role in removing active oxygen [26,27]. To investigate whether ME has antioxidative activity, the scavenging activity of ME on DPPH radical was measured. The DPPH radical scavenging activity of ME was found to be 26, 42, and 46% at ME concentrations of 50, 100, and 200 μg/mL, respectively (Figure 1). The antioxidative activity of ME showed a significant scavenging ability as compared with vehicle control. Therefore, these results indicate that ME has antioxidant properties. 

### 2.2. Effect of ME and Hydrogen Peroxide on Cell Viability of Pancreatic β-cells

**Figure 2:**
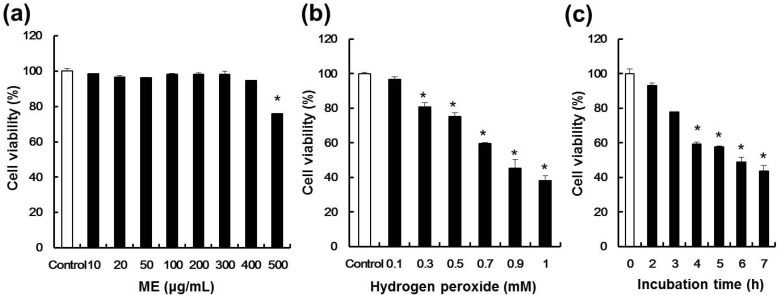
Viability of pancreatic MIN6N β-cells treated with ME and hydrogen peroxide. (**a**) Cell viability of pancreatic MIN6N β-cells treated with various concentrations of ME. (**b**) Cell viability of pancreatic MIN6N β-cells treated with various concentrations of hydrogen peroxide. (**c**) Cell viability of pancreatic MIN6N β-cells treated with different exposure time with 0.7 mM hydrogen peroxide. Data represent the mean ± SE of three independent experiments. Significance of the differences were compared with the control at * *p* < 0.05 by Student’s *t*-test.

To determine doses of ME that could be used without adversely affecting cell viability, the latter was determined using the MTT assay. The cells were seeded in 12-well plates and incubated for 24 h, after which different concentrations of ME were added for 20 h. As shown in Figure 2a, ME did not cause any cytotoxicity up to a concentration of 400 μg/mL, and no changes in cell morphology were observed by microscopic analysis (data not shown). Cell viability in pancreatic MIN6N β-cells at a concentration of 500 μg/mL was recorded as 76%. Thus, we conclude that ME could be used at concentrations up to 400 μg/mL in pancreatic β-cells in the follow-up studies described below. To measure the cytotoxicity of hydrogen peroxide, hydrogen peroxide (0.1–1.0 mM) was added to cultured cells for 4 h and cell viability was determined using the MTT assay (Figure 2b). Concentrations of hydrogen peroxide ≥ 0.7 mM dramatically reduced cell viability; for example, cell death reached ~60% after 0.7 mM hydrogen peroxide treatment. Moreover, treatment of cells with 0.7 mM hydrogen peroxide decreased cell viability in a time-dependent manner (Figure 2c). We therefore chose a hydrogen peroxide concentration of 0.7 mM and exposure time of 4 h, for all subsequent experiments.

### 2.3. Mulberry Extract Confers Protection Pancreatic β-Cells Against Hydrogen Peroxide-Induced Toxicity

**Figure 3:**
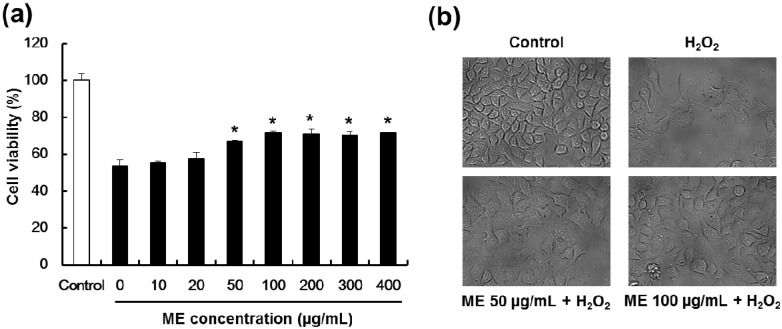
Protective effect of ME on hydrogen peroxide-treated pancreatic MIN6N β-cells. (**a**) Cell viability of pancreatic MIN6N β-cells treated with various concentrations of ME followed 0.7 mM hydrogen peroxide treatment. Data represent the mean ± SE of three independent experiments. Significance of the differences were compared with the hydrogen peroxide-treated group at * *p* < 0.05 by Student’s *t*-test. (**b**) Morphological characteristics of pancreatic MIN6N β-cells after various treatments. Morphology of MIN6N cells was observed by optical microscopy. Control, 0.7 mM hydrogen peroxide for 4 h, 0.7 mM hydrogen peroxide for 4 h with 50, and 100 μg/mL of ME, respectively. Magnification: 400×.

To evaluate whether ME could protect pancreatic β-cells against hydrogen peroxide-induced toxicity, cells were pre-treated with various concentrations of ME (from 10 to 400 μg/mL) for 20 h, followed by hydrogen peroxide treatment. Cell viability was measured using the MTT assay. In these experiments, cell viability decreased to 53.8% after hydrogen peroxide treatment in control cells. Pre-treatment with ME restored cell viability to 71.6% of control at a concentration of 400 μg/mL (Figure 3a). Morphologic changes induced by hydrogen peroxide were evaluated in order to quantify the protective effect of ME against hydrogen peroxide-induced cell damages. After hydrogen peroxide treatment, apoptosis-like morphologic changes such as shrinkage, detachment and cytoplasmic condensation were observed, and were inhibited in the presence of ME (Figure 3b). These results indicate that ME preserves both MIN6N β-cell viability and conserves the morphology of cells in the presence of hydrogen peroxide-induced oxidative stress.

### 2.4. ME Inhibited Hydrogen Peroxide-Induced ROS Generation and Lipid Peroxidation

**Figure 4:**
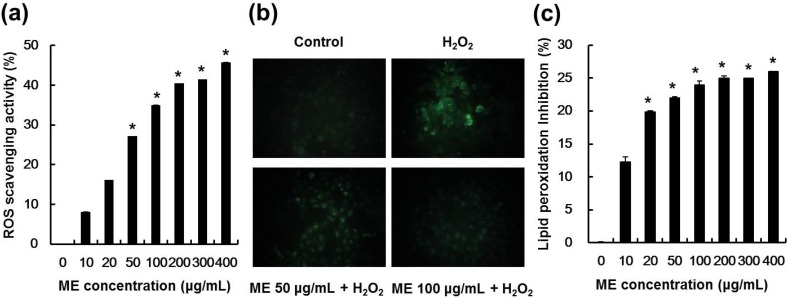
Effect of ME on hydrogen peroxide-induced ROS generation and lipid peroxidation in pancreaticMIN6N cells. (**a**) Intracellular ROS scavenging activity of ME. (**b**) Representative fluorescence images illustrate the increase in green fluorescence intensity of DCF produced by ROS in hydrogen peroxide-treated cells as compared to control and presence of ME. Magnification: 400×. (**c**) Lipid peroxidation inhibitory activity of ME. (**a** and **c**) Data represent the mean ± SE of three independent experiments. Significance of the differences were compared with the hydrogen peroxide-treated group at * *p* < 0.05 and by Student’s *t*-test.

To evaluate whether ME could inhibit hydrogen peroxide-induced intracellular ROS accumulation in pancreatic β-cells, cells were pre-treated with various concentrations of ME (from 10 to 400 μg/mL) for 20 h, followed by hydrogen peroxide treatment. Intracellular ROS levels in hydrogen peroxide-treated MIN6N β-cells were determined using the ROS-sensitive fluorescent probe H_2_DCF-DA, a cell-permeable dye that is cleaved by intracellular esterase into its non-fluorescent form, DCFH. DCFH can be further oxidized by hydrogen peroxide to form the fluorescent compound. The intracellular ROS scavenging activity of ME significantly increased in a dose-dependent manner and reached 34.5% at 100 μg/mL compared to the hydrogen peroxide-treated group (Figure 4a). Notably, the hydrogen peroxide-dependent increase in DCF green fluorescence intensity was dramatically decreased after treatment with 100 μg/mL ME, and remained similar to the level seen in control cells (Figure 4b). The inhibitory effect of ME on lipid peroxidation in hydrogen peroxide-treated MIN6N β-cells was determined by measuring thiobarbituric acid reactive substance (TBARS), a lipid peroxidation product [28]. As shown in Figure 4c, lipid peroxidation increased in cells exposed to hydrogen peroxide, whereas ME ameliorated lipid peroxidation. The inhibitory effect of ME was 24% at 100 μg/mL when compared to the ME-untreated group. These results indicate that ME possesses ROS scavenging activity suggesting ME would inhibit ROS-induced-cellular damage, such as lipid peroxidation, DNA condensation and/or fragmentation, and apoptotic cell death.

### 2.5. ME Inhibited Hydrogen Peroxide-Induced Apoptotic Cell Death

To evaluate whether or not the growth-inhibitory effect of hydrogen peroxide was associated with apoptosis, double-staining method using FITC-labeled annexin V and propidium iodine (PI) was performed. In undergoing early-apoptosis, phosphatidyl serine (PS) is exposed at the outer leaflet of plasma membrane. Annexin V binds specifically to PS allowing the discrimination between viable and apoptotic cells. Double staining the cells with annexin V and PI allowed us to detect apoptotic cells by flow cytometer. Among the four quadrants, upper left (UL) included cells that were stained negatively for Annexin V with being PI positive, and were considered necrosis cells. Upper right (UR) included cells that were both annexin V and PI positive, and were considered late-apoptotic cells. Lower left (LL) included cells that were stained negatively for both Annexin V and PI, and were considered undamaged cells. Lower right (LR) included cells that were stained with Annexin V with being PI negative, and were considered early-apoptotic cells [29]. 

**Figure 5:**
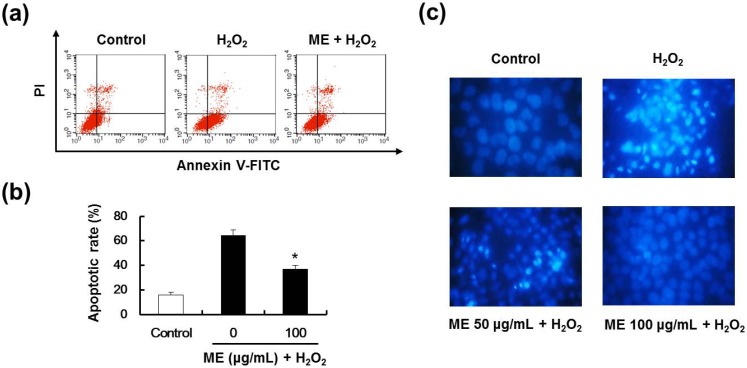
Effect of ME on hydrogen peroxide-induced apoptosis in pancreatic MIN6N cells. MIN6N β-cells were pre-incubated with different concentrations of ME for 20 h and then 0.7 mM hydrogen peroxide was added to allcells for 4 h, with the exception of the control group. (**a**) Apoptotic cells were detected by Annexin V and PI double staining and analyzed by flow cytometry. (**b**) The percentage of apoptotic-cell death from upper right plus lower right quadrants. Data represent the mean ± SE of three independent experiments. Significance of the differences were compared with the hydrogen peroxide-treated group at * *p* < 0.05 and by Student’s *t*-test. (**c**) Fluorescent assay of Hoechst 33342 staining. MIN6N β-cells were stained with Hoechst 33342 staining solution and visualized using a fluorescent microscope. Magnification: 400×.

Treatment with 0.7 mM hydrogen peroxide caused 60.78% of the cells to undergo early-apoptosis (Figure 5a,b). However, pre-treatment with 100 μg/mL ME dramatically inhibited hydrogen peroxide-induced early-apoptotic cell death (33.16%). In order to confirm the DNA condensation and/or fragmentation upon apoptosis induced by hydrogen peroxide, the chromatin of MIN6N β-cells was stained Hoechst 33342. As shown in Figure 5c, only hydrogen peroxide-treated MIN6N β-cells underwent DNA condensation and/or fragmentation. Conversely, these chromatin changes were reduced upon pre-treatment with 50 and 100 μg/mL ME. These results indicated that ME exhibited protective effects against oxidative stress-induced apoptosis in MIN6N β-cells.

## 3. Experimental

### 3.1. Materials

Mulberry fruit was purchased from a local company (Motgol Co., Pyeongchang, Korea). The mulberry fruits (15 kg) were extracted in 70% ethanol (36 L) for 24 h at room temperature three times. The solvent was filtered and evaporated under vacuum to afford 2,200 g of crude ethanol extract (yield, 14.6%). The obtained ethanol extract of mulberry fruits (ME) was kept at −20 °C until it was used for activity assessment. Dulbecco’s modified Eagle medium (DMEM), fetal bovine serum (FBS), penicillin-streptomycin (PS), and trypsin-EDTA were purchased from GIBCO (Grand Island, NY, USA). Dichlorodihydrofluorescein diacetate (H_2_DCF-DA) and the Apoptotic assay kit were obtained from Molecular Probes (Carlsbad, CA, USA). 2,2-diphenyl-1-picrylhydrazyl (DPPH), hydrogen peroxide (H_2_O_2_) and Hoechst 33342 were purchased from Sigma Biochemical (St. Louis, MO, USA). All other chemicals were analytical grade.

### 3.2. Cell Culture

The MIN6N pancreatic β-cells were derived from a mouse pancreatic islet cell line. The cells were provided by Professor H. Y. Kwon (College of Medicine, Hallym University, Chuncheon, Korea). MIN6N β-cells were cultured in DMEM (11 mM glucose) supplemented with 10% inactivated FBS and 1% penicillin/streptomycin and maintained at 37 °C in a humidified 5% CO_2_ incubator. The cells were cultured to ~85% confluence and harvested with 0.25% trypsin-EDTA. Cells were harvested and sub-cultured for an additional 48 h in DMEM. Cells were maintained in these culture conditions for all experiments.

### 3.3. DPPH Radical Scavenging Activity

For detection of DPPH radical, the method used by Blois [30] was adopted with suitable modifications. A solution of 150 μM DPPH in EtOH was used. The ME was dissolved in EtOH to obtain a concentration of 10–500 μg/mL. Each sample (400 μL each) was mixed with DPPH solution (600 μL) and incubated for 30 min to allow any reaction to occur. The content of residual DPPH was measured at 520 nm.

### 3.4. Cytotoxicity of ME

The effect of ME on the cytotoxicity for pancreatic MIN6N β-cells were evaluated using the (3-[4,5-dimethylthiazol-2-yl]-2,5-diphenyltetrazolium) bromide (MTT) assay, as described previously [31]. Cells were seeded in 12-well plates, incubated for 24 h, treated with ME, and then incubated for a further 20 h at 37 °C. MTT solution (500 μg/mL) was then added to each well and incubated for 3 h at 37 °C. The formazan crystal in each well was dissolved in isopropyl alcohol and absorbance of each well was then measure at 595 nm using an ELISA microplate reader (model 550, Bio-Rad, Hercules, CA, USA).

### 3.5. Protective Effect of ME and Image Analysis

To investigate the protective effect of ME in hydrogen peroxide-induced cell death, the cells were incubated with DMEM containing 0.5% FBS for 24 h prior to incubation with indicated dose of ME for 20 h and then treated with 0.7 mM hydrogen peroxide for 4 h. Cell viability was measured by means of the MTT assay as described above. Morphological observation of the cells was performed using an Eclipse 50 microscope (Nikon, Tokyo, Japan).

### 3.6. Intracellular ROS Scavenging Activity and Image Analysis

To determine the effect of ME on hydrogen peroxide-induced ROS generation, MIN6N β-cells were seeded the day before ME treatment in 12-well plates. Cells were treated with various concentrations of ME for 20 h, followed by the addition of 0.7 mM hydrogen peroxide to each well. After 4 h incubation, 5 μM H_2_DCF-DA solution in phosphate buffered saline (PBS, pH 7.38) was added, and the fluorescence was measured at excitation and emission wavelengths of 485 nm and 535 nm, respectively, using a microplate spectrofluorometer. For image analysis of the production of intracellular ROS, MIN6N β-cells were seeded in coverslip-loaded 12-well plates, which were then treated with various concentrations of ME for 20 h, followed by the addition of 0.7 mM hydrogen peroxide to each well. After 4 h of incubation, H_2_DCF-DA solution was added to each well of the plate, which was incubated for 2 h at 37 °C. Images of the stained cells were collected using a fluorescence microscope (Nikon, Tokyo, Japan).

### 3.7. Lipid Peroxidation Inhibitory Activity

Lipid peroxidation was assayed using the thiobarbituric acid (TBA) reaction [32]. Briefly, MIN6N β-cells were seeded in 12-well plates and incubated for 24 h. Then, cells were treated with various concentrations of ME for 20 h, followed by the addition of 0.7 mM hydrogen peroxide to each well. After 4 h incubation, the cells were washed cold PBS, harvested with 0.25% trypsin-EDTA, and homogenized in cold 1.15% KCl. A 100 μL aliquot of homogenized cells were then mixed with 0.2 mL of 8.1% sodium dodecyl sulfate, 1.5 mL of 20% acetic acid (pH 3.6), and 1.5 mL of 0.8% TBA. The mixture was then heated to 95 °C for 2 h. After cooling to RT, an n-butanol/pyridine mixture (15:1, v/v) was added and then the mixture was shaken for 5 min before centrifugation at 1,000 g for 10 min. The supernatant was isolated, and the absorbance was measured at 535 nm.

### 3.8. Flow Cytometric Analysis

Cells undergoing apoptosis were examined using a FITC-labeled Annexin V/propidium iodide (PI) apoptosis detection kit (Molecular Probes, Eugene, OR, USA) according to the manufacturer’s instructions. In briefly, cells were harvested by trypsinization, washed with phosphate-buffer solution, and centrifuged to collect the cell pellet. The number of cells was adjusted to 1 × 10^6^ cells/mL. Cells were then resuspended in binding buffer (10 mM HEPES, 140 mM NaCl, 2.5 mM CaCl_2_, pH 7.4) and stained with FITC-labeled Annexin V/PI at RT for 15 min in light-protected conditions. Flow cytometric analysis was performed using a FACSCalibur flow cytometer (Becton Dickinson, Mountain View, CA, USA) within 1 h after supravital staining. FITC-labeled Annexin V was analyzed using excitation and emission settings of 488 nm and 535 nm, respectively. PI was analyzed using excitation and emission setting of 488 nm and 575 nm, respectively. During each run, 10,000 cells were measured. The percentages of cells were calculated by Cell Quest software (Becton Dickinson). Early apoptotic-cells were Annexin V-positive and PI-negative; however, late-apoptotic cells were positive for both Annexin V and PI. The apoptotic index (%) was calculated as the sum of early and late-apoptotic cells divided by the total number of events.

### 3.9. Measurement of DNA Condensation and/or Fragmentation

MIN6N β-cells were labeled using the cell-permeable, DNA specific fluorescent dye Hoechst 33342 [33]. Cells with homogeneously stained nuclei were considered viable, whereas presence of chromatin condensation and/or fragmentation was indicative of apoptosis [34]. The MIN6N β-cells were seeded in 12-well plates. Then, 24 h after plating, the cells were treated with various concentrations of ME for 20 h, followed by further incubation for 4 h prior to exposure to 0.7 mM hydrogen peroxide. Then, Hoechst 33342 (stock 10 mg/mL) was added to each well, followed by 15 min of incubation at room temperature. Images of the stained cells were collected using a fluorescence microscope (Nikon), in order to examine the degree of DNA condensation and/or fragmentation.

### 3.10. Statistical Analysis

All measurements were from at least three independent experiments and values were expressed as means ± SD. Statistical analysis was performed using Student’s *t*-test. A value of *p* < 0.05 was considered significant.

## 4. Conclusions

In this study, we used hydrogen peroxide-induced toxicity of pancreatic β-cells as a model to evaluate the antioxidant activity of ME, and identified the appropriate concentrations that elicited the greatest protective effects. Furthermore, ME pre-treatment reduced the amount of ROS in MIN6N cells treated with hydrogen peroxide. In addition, cells exposed to hydrogen peroxide exhibited distinct morphological features of apoptosis, such as membrane blebbing and an increase in annexin V staining. However, cells that were pretreated with ME had a significantly reduced percentage of early-apoptotic cells, as shown by morphological changes and the reduction in annexin V staining. These results demonstrate that mulberry protects the β-cells of the pancreas by limiting ROS generation, and provides evidence to support the use of mulberry as a preventative treatment for diabetes. The anti-apoptotic signaling mechanism of mulberry-derived active compound should be further investigated as a natural remedy for diabetes treatment.
